# Closing Editorial: Advancements in Artificial Intelligence for Dentomaxillofacial Radiology—Current Trends and Future Directions

**DOI:** 10.3390/diagnostics15101222

**Published:** 2025-05-13

**Authors:** Kaan Orhan, Andre Luiz Ferreira Costa, Sérgio Lúcio Pereira de Castro Lopes

**Affiliations:** 1 Department of Dentomaxillofacial Radiology, Faculty of Dentistry, Ankara University, Ankara 06560, Turkey; 2 Department of Dental and Maxillofacial Radiodiagnostics, Medical University of Lublin, 20-059 Lublin, Poland; 3 Medical Design Application and Research Center (MEDITAM), Ankara University, Ankara 06000, Turkey; 4 Postgraduate Program in Dentistry, Cruzeiro do Sul University (UNICSUL), São Paulo 08060-070, SP, Brazil; alfcosta@gmail.com; 5 Department of Diagnosis and Surgery, São José dos Campos School of Dentistry, São Paulo State University (UNESP), São José dos Campos 12245-000, SP, Brazil; sergio.lopes@unesp.br

Artificial intelligence (AI) continues to redefine diagnostic approaches across medical disciplines, and its impact on dentomaxillofacial radiology has increased exponentially in recent years. With advances in deep learning (DL), large annotated datasets, and computational power, AI has become a powerful tool in analyzing complex craniofacial imaging data, offering precision, speed, and reproducibility beyond human capacity in many contexts.

This Special Issue of *Diagnostics*, entitled “Advancements in Artificial Intelligence for Dentomaxillofacial Radiology”, sought to capture this dynamic shift by curating original research and reviews that span the spectrum of AI applications in dental imaging—from traditional 2D panoramic radiography to 3D CBCT and emerging dental MRI technologies.

A key focus of this Issue was on algorithmic performance in real-world diagnostic scenarios. Contributions employing YOLOv8 and similar architectures demonstrated the near-perfect detection of critical anatomical structures, including the mandibular canal, condyles, and dental implants, across variable image qualities and devices. These studies reflect a crucial trend toward the development of device-agnostic AI models with clinically reliable precision. For instance, George et al. (2023) analyzed panoramic dental radiographs using the YOLOv8 model and examined its effectiveness for diagnosis [1].

Segmentation-based models also featured prominently. U-Net, nnU-Net, and U^2^-Net-based frameworks were used to delineate pulp chambers, periapical lesions, and maxillofacial bones, yielding Dice Similarity Coefficients (DSCs) above 0.90 in most cases. These models not only enhance reproducibility but also facilitate downstream applications such as volumetric assessment, AI-assisted treatment planning, and augmented surgical navigation. For example, Baydar et al. (2023) utilized a U-Net architecture to evaluate dental bite-wing radiographs, achieving high accuracy in detecting various dental conditions [2].

Of particular interest are the articles examining explainability—a growing imperative in AI-driven diagnostics. Black-box models, while powerful, face resistance in clinical implementation, being without interpretability. Research using Grad-CAM, SHAP, and attention maps demonstrated how AI can transparently highlight diagnostic cues, thereby improving clinician trust and legal defensibility. A scoping review by Ghosh et al. (2023) emphasized the importance of interpretability and explainability in medical AI applications, highlighting the need for transparent models in clinical settings [3].

Despite these strides, several barriers remain. First, model generalizability is often limited by dataset homogeneity. Even the most promising models can falter when applied to imaging protocols or populations not represented in the training data. Second, integration into clinical workflow requires alignment with standards like DICOM, PACS systems, and regulatory approval pathways (e.g., CE/FDA clearances). Third, few studies have addressed longitudinal AI model performance or outcomes-based validation in dental radiology—critical aspects for widespread adoption [4,5,6,7,8,9,10,11].

Future research should aim to achieve the following objectives:

- Multimodal fusion—combining radiographic, intraoral, and clinical data for holistic AI-based diagnostics [8].
- Real-time integration—deploying AI tools at the point of care, especially in underserved areas or during tele-dentistry sessions [9].
- Texture analysis as a valuable technique in dentomaxillofacial diagnosis, providing an advanced method for quantification and characterization of different image modalities [10].
- Ethical AI frameworks—ensuring bias mitigation, privacy preservation, and transparent model auditing across global dental populations [11].

This Special Issue was enriched by contributions from researchers across multiple continents, reflecting the global relevance of AI in oral and maxillofacial imaging. The breadth of work underscores both the maturity and future potential of this interdisciplinary field. We thank all authors, reviewers, and editorial staff for their efforts and encourage readers to build on the strong foundation laid by this collection.

We hope the studies here serve as a catalyst for translational research and encourage the deeper integration of AI into dental radiology education, clinical care, and policy-making [4,5,6,7,8,9,10,11].
